# Editorial: New perspectives on the role of sensory feedback in speech production

**DOI:** 10.3389/fnhum.2023.1189751

**Published:** 2023-05-09

**Authors:** John F. Houde, Lucie Ménard, Jeffery A. Jones, Douglas M. Shiller, Xing Tian

**Affiliations:** ^1^Department of Otolaryngology – Head and Neck Surgery, University of California San Francisco, San Francisco, CA, United States; ^2^Département de Linguistique, Université du Québec à Montréal, Montréal, QC, Canada; ^3^Department of Psychology, Wilfrid Laurier University, Waterloo, ON, Canada; ^4^École d'orthophonie et d'audiologie, Faculté de médecine, Université de Montréal, Montréal, QC, Canada; ^5^Department of Neural and Cognitive Sciences, NYU Shanghai, Shanghai, China; ^6^NYU-ECNU Institute of Brain and Cognitive Science at NYU Shanghai, Shanghai, China; ^7^Shanghai Key Laboratory of Brain Functional Genomics (Ministry of Education), School of Psychology and Cognitive Science, East China Normal University, Shanghai, China

**Keywords:** speech production, speech perception, speech motor control, speech feedback monitoring, speech development, sensory feedback

Studies on the role of sensory feedback in speech production have revealed much about sensorimotor integration mechanisms in speech-motor control. These studies have a rich history dating back over a century, starting with Lombard's (1911) work on the impact of noise on speech loudness. Recent advancements in technology and techniques have greatly accelerated the progress of this field. In this Special Topic, our aim was to bring together a collection of cutting-edge studies that reflect the exciting new directions and breakthroughs in this area of research, particularly over the past few years.

The study by Oschkinat et al. adds greatly to our understanding of the role of sensory feedback in the timing of speech production. They used focal distortions of the duration of consonant-vowel-consonant syllables in speakers' auditory feedback and showed that speakers adapted to distortions of vowel duration but only adapted to distortions in consonant duration when the consonant was in the coda position. Additionally, Oschkinat et al. found that high sensitivity in rhythm and interval perception, along with high variability in rhythm and interval production, was correlated with the degree of adaptation observed in speakers. These findings offer valuable insights into the mechanisms used by the auditory system to monitor and adjust speech timing, which may have implications for the development of speech rehabilitation techniques.

The role of feedback in speech timing is also addressed in a new synthesis by Tilsen. Tilsen proposes a framework consisting of a palette of “time responders” (TiRs) that represent the ways in which feedback (both internal and external) could control the timing of utterance production. TiRs can be combined to govern gestural timing within utterances and utterance sequencing. They also form the basis of the hypothesis that speakers change their speech rate by changing how they attend to sensory feedback as they speak.

Speech scientists have long worked to understand speech variability and stability. The study by Wang and Max demonstrated that speakers actively control their speech variability by exposing them to auditory feedback alterations that either magnified or attenuated their perceived errors in producing vowels. Attenuation caused speakers to gradually increase their variability over repeated productions. Nault et al. investigated the effect of feedback variability on speech stability, revealing that speakers adapt only to consistent changes in their auditory feedback. Their work suggests that the consistency of feedback facilitates the stability of speech sensorimotor control.

Advances in neuroimaging have also greatly facilitated our understanding of how sensory feedback is processed during speaking. Recent research has demonstrated how this process is compromised in dysfunctional conditions, such as stuttering. The study by Garnett et al. is a noteworthy example, offering further evidence of the relationship between stuttering and abnormal auditory feedback processing. Additionally, this study suggests that stuttering may be linked to disruptions in speech sensorimotor function by the default mode network.

Some of the studies included in this Research Topic focus on speech perception, which sensory feedback mechanisms likely depend on. Goldenberg et al. provide further support for the findings of Gick and Derrick (2009), showing that air puffs, even on the hands, can influence the perception of ambiguous consonant sounds toward voiceless consonants. Johnson et al. found a correlation between the right-hemisphere auditory cortical speech responses and the likelihood of study participants experiencing auditory hallucinations.

One key question about sensory feedback is how its role in speaking evolves during development. To address this question, the article by Coughler et al. provides a comprehensive review of pediatric responses to altered auditory feedback. The studies they review show that while children have prolonged response times to auditory feedback perturbations, by the age of four they display sensorimotor adaptation that is qualitatively similar to adults. However, it is noted that the limited number of studies on this subject makes it difficult to draw definitive conclusions, underlining the need to explore more fully the plasticity of sensory feedback control of speaking across the lifespan.

Recently, researchers have developed various new models that help to explain the role of feedback in the development of speech production. One such model, proposed by Kröger et al., provides a comprehensive account of speech production by postulating an evolving role for sensory feedback during development. In this model, sensory feedback initially plays a crucial role in an undirected babbling process, creating internalized sensory-motor relationships. These relationships are then used when children attempt to imitate words produced by others. During this process, they initially select motor states that were previously associated with the sounds of the target utterance and then vary them until they receive feedback that their speech has been understood.

Another model, proposed by Davis and Redford, describes a dual-lexicon model of speech-motor planning that evolves continuously with experience from childhood through adulthood. According to their model, words have perceptual representations (exemplars) that evolve as the speaker hears the speech of others as well as auditory feedback of their own word productions. In addition, words have motor representations (silhouettes) that evolve as the speaker plans word productions. This process balances matching the target perceptual exemplar with articulatory ease and prior motor habits.

The final theme covered in this Research Topic is determining how sensory feedback processing varies across speakers. Kearney et al. propose a unique approach in which they fit the timecourse of a speaker's response to auditory pitch feedback perturbations to a simplified version of the DIVA model. The authors find that pitch perturbation responses vary across speakers but remain consistent within each individual, creating a distinct “fingerprint” of their speech motor system. If such fingerprints can be expressed in interpretable parameters, the authors suggest that the effects of disease states on the pitch perturbation reflex can be similarly expressed as meaningful changes in these interpretable parameters.

This marks the end of a brief overview of the papers on this Research Topic. It offers a general idea of the topics covered but may generate further questions. We encourage you to delve deeper by reading the individual papers, which offer a more comprehensive examination of this fascinating area of research.

## Author contributions

JH wrote the initial draft. JH, LM, JJ, DS, and XT contributed to the writing/editing of manuscript. All authors contributed to the article and approved the submitted version.
